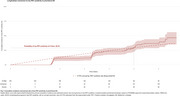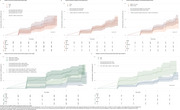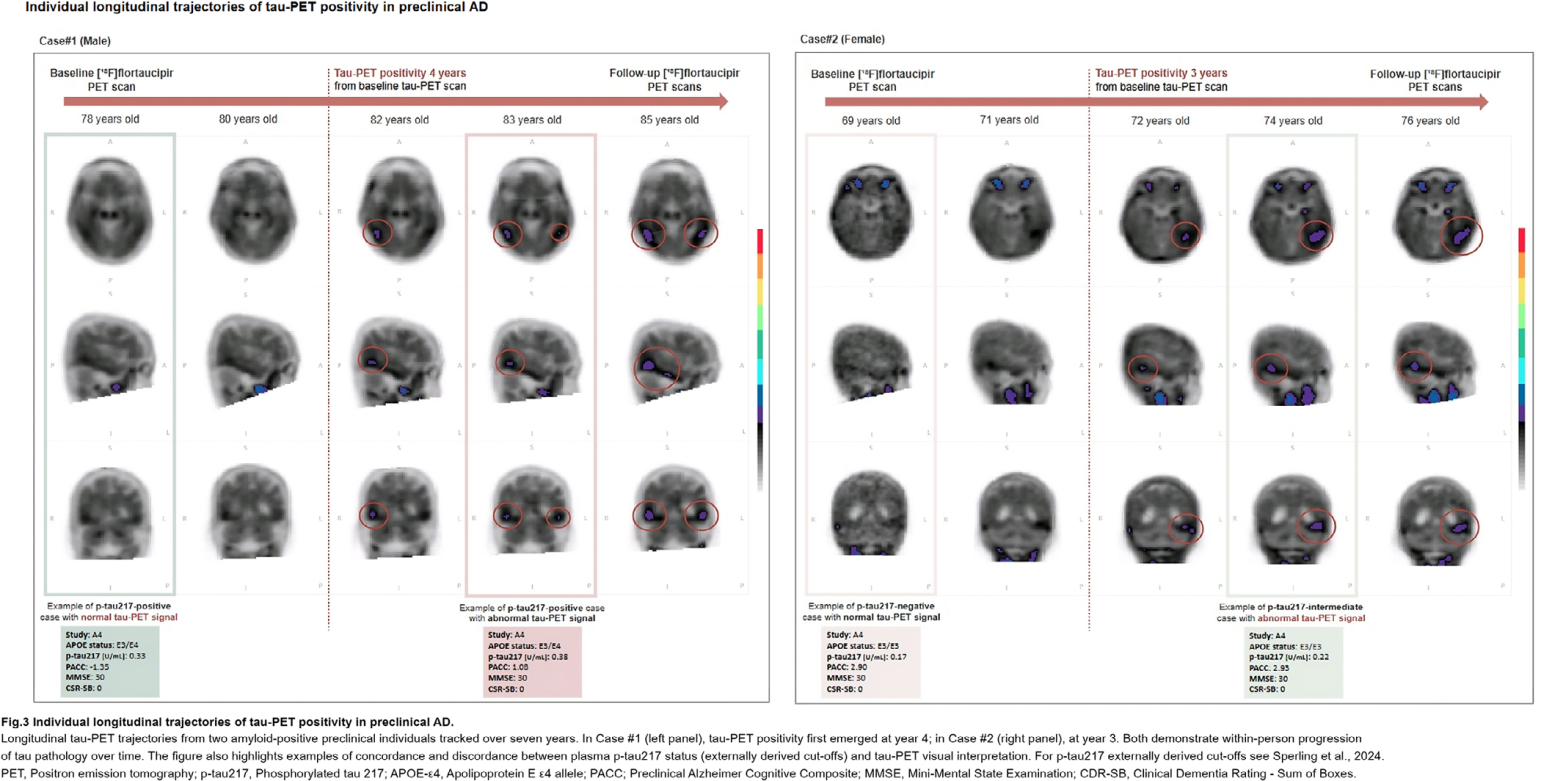# Rates of Progression to Tau‐PET Positivity in Preclinical Alzheimer’s Disease

**DOI:** 10.1002/alz70861_108962

**Published:** 2025-12-23

**Authors:** Stamatia Karagianni, David M Cash, Anxo M. Minguillón Pereiro, Jesús Silva‐Rodríguez, Michel J. Grothe, Pablo Aguiar, Michael Schöll, Alexis Moscoso Rial

**Affiliations:** ^1^ Wallenberg Centre for Molecular and Translational Medicine, University of Gothenburg, Gothenburg Sweden; ^2^ Department of Psychiatry and Neurochemistry, Institute of Neuroscience and Physiology, The Sahlgrenska Academy, University of Gothenburg, Gothenburg Sweden; ^3^ Dementia Research Centre, Department of Neurodegenerative Disease, UCL Queen Square Institute of Neurology, University College London, London UK; ^4^ Complexo Hospitalario Universitario Santiago de Compostela, Santiago de Compostela, Travesía da Choupana Spain; ^5^ CIEN Foundation, Reina Sofia Alzheimer Center, ISCIII, Madrid, Madrid Spain; ^6^ Faculty of Medicine and Center for Research in Molecular Medicine and Chronic Diseases (CIMUS), University of Santiago de Compostela, Santiago de Compostela, Travesía da Choupana Spain; ^7^ Department of Neuropsychiatry, Sahlgrenska University Hospital, Gothenburg Sweden; ^8^ Dementia Research Centre, Queen Square Institute of Neurology, University College London, London UK; ^9^ Instituto de Investigación Sanitaria de Santiago de Compostela, Santiago de Compostela, Travesía da Choupana s/n Spain

## Abstract

**Background:**

Previous work from our group has shown that tau‐PET positivity—defined using an FDA/EMA‐approved visual interpretation method reflecting Braak stages V–VI—marks a key milestone closely associated with the onset of clinical symptoms in preclinical Alzheimer’s disease (AD). Understanding progression rates to tau‐PET positivity is crucial for designing prevention trials, evaluating tau‐PET as an outcome measure, and clarifying the clinical significance of amyloid‐β (Aβ) positivity in the absence of PET‐detectable tau pathology. Here, we estimate progression rates to visually assessed tau‐PET positivity and investigate associated factors and biomarkers.

**Method:**

We included 371 Aβ‐positive cognitively unimpaired (CU) participants from the A4 Study who underwent [^18^F]flortaucipir PET imaging. Plasma *p* ‐tau217 (MesoScale ECL assay) was available for 355 participants. A trained reader rated the [^18^F]flortaucipir scans using an FDA/EMA‐approved visual method reflecting Braak stages V–VI. Kaplan‐Meier and exponential models were used to estimate cumulative incidence and annual progression rates. Cox proportional hazards models assessed the influence of age, sex, APOE‐ε4 genotype, plasma *p* ‐tau217 status, and baseline medial temporal lobe (MTL) tau‐PET signal. Plasma *p* ‐tau217 classification used externally derived two‐tier cut‐offs (Sperling et al., 2024), and baseline MTL signal was quantified using CenTauR (2 CTR_z_) thresholds (Villemagne et al., 2023).

**Result:**

Out of the total 371 Aβ‐positive CU, 253 had a negative baseline tau‐PET scan. After five years, 29.2% of these participants progressed tau‐PET positivity, with an estimated annual incidence of 6.75% (Figure 1). Age, sex, and APOE‐ε4 status were not associated with progression (HR=1.01, *p* =0.7; HR=1.05, *p* =0.8; HR=1.21, *p* =0.5) (Figure 2A‐C). Plasma *p* ‐tau217 stratified individuals by risk (HR=3.06 positive vs. negative, *p* =0.001) (Figure 2D). Elevated baseline MTL tau‐PET signal was associated with future progression (HR=2.41, *p* =0.01) (Figure 2E). Qualitative analyses over seven years revealed clear tau‐PET progression, with initial accumulation often outside the MTL and notable discordance between plasma *p* ‐tau217 and tau‐PET in some cases (Figure 3).

**Conclusion:**

Only one in three Aβ‐positive CU individuals with a negative tau‐PET scan will progress to tau‐PET positivity within five years. Combining blood‐based and imaging biomarkers may improve identification of individuals at higher risk for tau accumulation, which may inform future prevention trial design.